# During postnatal development endogenous neurosteroids influence GABA-ergic neurotransmission of mouse cortical neurons

**DOI:** 10.1016/j.neuropharm.2015.11.019

**Published:** 2016-04

**Authors:** Adam R. Brown, Scott J. Mitchell, Dianne R. Peden, Murray B. Herd, Mohsen Seifi, Jerome D. Swinny, Delia Belelli, Jeremy J. Lambert

**Affiliations:** a Division of Neuroscience, Medical Research Institute, Ninewells Hospital & Medical School, Dundee University, Dundee, UK; b Institute for Biomedical and Biomolecular Sciences, School of Pharmacy and Biomedical Sciences, University of Portsmouth, Portsmouth, UK

**Keywords:** GABA_A_ receptor, Neurosteroid, Neonatal development, Cortex

## Abstract

As neuronal development progresses, GABAergic synaptic transmission undergoes a defined program of reconfiguration. For example, GABA_A_ receptor (GABA_A_R)-mediated synaptic currents, (miniature inhibitory postsynaptic currents; mIPSCs), which initially exhibit a relatively slow decay phase, become progressively reduced in duration, thereby supporting the temporal resolution required for mature network activity. Here we report that during postnatal development of cortical layer 2/3 pyramidal neurons, GABA_A_R-mediated phasic inhibition is influenced by a resident neurosteroid tone, which wanes in the second postnatal week, resulting in the brief phasic events characteristic of mature neuronal signalling. Treatment of cortical slices with the immediate precursor of 5α-pregnan-3α-ol-20-one (5α3α), the GABA_A_R-inactive 5α-dihydroprogesterone, (5α-DHP), greatly prolonged the mIPSCs of P20 pyramidal neurons, demonstrating these more mature neurons retain the capacity to synthesize GABA_A_R-active neurosteroids, but now lack the endogenous steroid substrate. Previously, such developmental plasticity of phasic inhibition was ascribed to the expression of synaptic GABA_A_Rs incorporating the α1 subunit. However, the duration of mIPSCs recorded from L2/3 cortical neurons derived from α1 subunit deleted mice, were similarly under the developmental influence of a neurosteroid tone. In addition to principal cells, synaptic GABA_A_Rs of L2/3 interneurons were modulated by native neurosteroids in a development-dependent manner. In summary, local neurosteroids influence synaptic transmission during a crucial period of cortical neurodevelopment, findings which may be of importance for establishing normal network connectivity.

## Highlights

• Upon postnatal maturation GABAA receptor synaptic inhibition is reduced in duration.
• Reduced synthesis of local neurosteroids contributes to this cortical plasticity.
• The study reveals a potent mechanism to locally regulate cortical neuron activity.

## Abbreviations

α1^−/−^: GABA_A_R α1 subunit “knockout”.
5α3α: 5α-pregnan-3α-ol-20-one; allopregnanolone.
5α-DHP: 5α-dihydroprogesterone, or 5α-pregnane-3,20-dione.
5α-R: 5α-reductase.
CD: cyclodextrin
τ_w_: weighted decay time constant of mIPSC decay.
aCSF: artificial cerebrospinal fluid.
ANOVA: analysis of variance statistical test.
DMSO: dimethylsulphoxide.
ECS: extracellular solution.
GABA: γ-aminobutyric acid.
GABA_A_R: γ-aminobutyric acid type A receptor.
GAD67-GFP: glutamic acid decarboxylase-green fluorescent protein.
ICS: intracellular solution.
KS: Kolmogorov–Smirnov statistical test.
L2/3: cortical layer 2/3.
mIPSC: miniature inhibitory postsynaptic current.
P: postnatal day.
S.E.M: standard error of the mean.
T50: time taken for mIPSCs to decay from peak amplitude by 50%.
TTX: tetrodotoxin.
VB: ventrobasal.

## 1 Introduction

The postnatal brain undergoes considerable neuronal plasticity to meet the changing demands of rapidly developing networks. During this critical time the duration of synaptic events mediated by GABA_A_Rs becomes progressively reduced, permitting postsynaptic neurons to respond to input from certain fast-spiking GABA-ergic interneurons and thereby appropriately influence the temporal window for postsynaptic excitation (Whittington et al., 2011, Deidda et al., 2014, Fritschy and Panzanelli, 2014). Alterations to the subunit composition of synaptic GABA_A_Rs are implicated in producing these crucial changes to inhibitory postsynaptic current (IPSC) kinetics (Brickley et al., 1996, Okada et al., 2000, Vicini et al., 2001, Juttner et al., 2001, Goldstein et al., 2002, Bosman et al., 2005; Takahashi, 2005, Fritschy and Panzanelli, 2014, Deidda et al., 2014). GABA_A_Rs are members of the Cys-loop transmitter-gated ion channel family and in common with glycine, nicotinic acetylcholine and 5HT_3_ receptors are composed of five subunits (Olsen and Sieghart, 2008). In mammals 19 subunit genes underpin the expression of ∼20–30 native GABA_A_R subtypes, which display distinct pharmacological and physiological properties (Olsen and Sieghart, 2008). In the CNS, these GABA_A_R subtypes exhibit a heterogeneous expression pattern, which importantly in many neurons is known to change during neonatal development (Olsen and Sieghart, 2008, Fritschy and Panzanelli, 2014, Rudolph and Mohler, 2014). In particular, an increased expression of receptors incorporating the α1 subunit (α1-GABA_A_Rs) is implicated in the appearance of short duration IPSCs (Okada et al., 2000, Vicini et al., 2001, Peden et al., 2008, Eyre et al., 2012, Deidda et al., 2014, Fritschy and Panzanelli, 2014). However, during development of thalamocortical inhibitory synapses, changes to IPSC kinetics occur prior to the temporal expression of the α1 subunit (Peden et al., 2008, Brown et al., 2015), implicating, at least in these neurons, additional factor(s) that influence GABA_A_R ion channel gating properties.

Certain naturally occurring neurosteroids act in a non-genomic manner as endogenous positive allosteric modulators of the GABA_A_R (Belelli and Lambert, 2005, Zorumski et al., 2013). The cortical levels of these neurosteroids change during neonatal development (Grobin and Morrow, 2001). Furthermore, the enzymes required to synthesize these GABA_A_R-active steroids are expressed in certain neurons, suggesting that these local neuromodulators may act as paracrine, or autocrine messengers, to locally influence neuronal inhibition (Agis-Balboa et al., 2006, Do Rego et al., 2009, Castelli et al., 2013, Brown et al., 2015). Here, we demonstrate for mouse cortical L2/3 pyramidal neurons and interneurons that during early (P7-15) neonatal development, their synaptic GABA_A_Rs are influenced by an endogenous neurosteroid tone, which consequently prolongs the duration of phasic GABAergic neurotransmission. During subsequent development this modulation wanes, such that by P20-24 it has dissipated, resulting in brief IPSCs, characteristic of mature inhibitory synapses. However, when provided with 5α-dihydroprogesterone (5α-DHP), the 5α3α precursor, these more mature neurons retain the capacity to synthesise GABA_A_R-active neurosteroids, suggesting that the developmental changes to GABAergic neurotransmission reflect a timed loss of steroid substrate, acting in concert with the established ontogenetic pattern of α1 subunit expression. Importantly, neurosteroid levels are not static, but are perturbed in a variety of physiological and pathophysiological conditions (Belelli and Lambert, 2005, Zorumski et al., 2013). Therefore, given the role GABA_A_Rs may play in a number of disorders including autism, schizophrenia, Fragile X and Down syndrome (Deidda et al., 2014, Rudolph and Mohler, 2014), these findings may not only be important in better understanding how phasic GABAergic neurotransmission changes to accommodate the demands of neuronal network activity during development, but may additionally allow new insights into the pathology of certain neurodevelopmental disorders.

## 2 Materials & methods

### 2.1 Breeding of mice

All animal studies were approved by the University of Dundee Ethical Review Committee (Home Office Project Licenses 60/4005 and 70/8161, Dr. Belelli), and complied with Schedule 1 of the UK Government Animals (Scientific Procedures) Act, 1986. Transgenic α1 subunit ‘knockout’ (α1^−/−^) mice were generated on a mixed C57BL6-129SvEv background (Sur et al. 2001). Transgenic GAD 67-GFP “knock-in” mice were generated on a C57BL/6J background as described previously (Tamamaki et al. 2003). Electrophysiological experiments were performed on brain slices prepared from the first 2–3 generations of α1^−/−^, GAD67-GFP, or corresponding WT offspring from heterozygous (^+/−^) breeding pairs housed at the University of Dundee.

### 2.2 Preparation of brain slices for electrophysiology

Cortical slices were prepared from postnatal day (P) P7 - 24 WT, α1^−/−^, or GAD 67-GFP mice of either sex. Mice were killed by cervical dislocation, the brain dissected and placed in ice-cold oxygenated (95% O_2_/5%CO_2_) artificial cerebrospinal fluid (aCSF) containing (in mM): 225 sucrose, 2.95 KCl, 1.25 NaH_2_PO_4_, 26 NaHCO_3_, 0.5 CaCl_2_, 10 MgSO_4_, 10 glucose, (pH 7.4; 328–330 mOsm). The brain was sectioned in the coronal plane using a Vibratome series 1000 PLUS Sectioning System (Intracell, Royston, Hertfordshire, UK). Slices were cut at 300–350 μm thickness for mice of P15, or older, and 400 μm, for younger animals. Slices were immediately transferred on to a nylon mesh platform housed within a chamber containing circulating oxygenated extracellular solution (ECS, in mM: 126 NaCl, 26 NaHCO_3_, 2.95 KCl, 1.25 NaH_2_PO_4_, 2 MgCl_2_, 2 CaCl_2_, 10 glucose [306–309 mOsm]) and allowed to rest at room temperature for a minimum of 1 h before electrophysiological recording.

### 2.3 Voltage-clamp recording

During recording, cortical slices were perfused with ECS maintained at 35 °C using a gravity based perfusion system set to a flow rate of 3–5 ml/min and recycled to a 50 ml oxygenated reservoir using a peristaltic pump (Minipuls 3, Gilson, UK). Intracellular solution (ICS) containing (in mM): 135 CsCl, 10 HEPES, 10 EGTA, 2 MgCl_2_, 1 CaCl_2_, 2 Mg-ATP and 5 QX-314 (pH 7.2–7.3, 290–300 mOsm) was used for whole-cell recording. Patch pipettes were pulled from thick-walled borosilicate glass (0.95 mm I.D. 1.55 mm E.D. Garner Glass Co. Claremont, CA), using a Narashige PC-10 electrode puller (Narashige, Japan). When filled with the above ICS, pipettes with an open tip resistance of 2–6 MΩ were obtained. Neurons were visually identified for investigation using an upright Olympus BX50WI microscope (Olympus, Southall, UK) equipped with IR-DIC optics. Pyramidal neurons located within cortical L2/3 were identified based on their canonical pyramidal morphology. L2/3 GABAergic interneurons were identified in cortical slices derived from GAD67-GFP “knock-in” mice using epifluorescence microscopy. Neurons were voltage-clamped at −60 mV using an Axopatch 1D amplifier (Molecular Devices, CA, USA) and filtered at 2 kHz. GABA_A_R-mediated mIPSCs were isolated by supplementing the ECS with kynurenic acid (2 mM), tetrodotoxin (TTX, 500 nM) and strychnine (1 μM). Data was acquired and digitised (10 kHz) using a NIDAQ mx card (National Instruments, TX, USA) and stored directly to PC using WinEDR software (Strathclyde University, UK). Series resistance compensation was applied up to 80%. Recordings were omitted from analysis if the series resistance changed by more than 20% during the experiment, or if they exceeded 15 MΩ.

### 2.4 Drugs and reagents

For *in vitro* experiments, finasteride, indomethacin, 5α3α and 5α-DHP were prepared as concentrated stock solutions (1000x final concentration) in DMSO, whereas bicuculline methobromide and TTX were prepared as concentrated stock solutions in distilled water. Drug stock solutions were diluted to the final required concentration in ECS, whereas kynurenic acid was dissolved directly into the ECS. Similarly, α-CD and γ-CD were dissolved directly into the extracellular and intracellular solution.

For acute studies with 5α3α (1 μM), the steroid was perfused directly in to the recording chamber, with the effect determined on mIPSCs acquired after ∼ 7 min of drug contact with the slice preparation. To investigate the impact of prolonged exposure to either 5α3α (100 nM), or 5α-DHP (3 μM), the test steroid was pre-incubated with the cortical slice at room temperature for > 2 h, before the tissue was transferred to the recording chamber, where it was continuously perfused with ECS (see Section 2.3 above) containing the test steroid. Note for some experiments with 5α-DHP (3 μM) the cortical slice was co-incubated with indomethacin (100 μM). The CD studies employed two protocols: the first involved pre-incubating cortical slices in the holding chamber at room temperature with either α-, or γ-CD (1 mM, >1 h). Recordings were then made with both ECS and ICS containing the CD (1 and 0.5 mM, respectively). In the second protocol, CD was included only in the recording pipette (0.5 mM). When the CD was applied to the intracellular compartment alone, mIPSCs were only included for analysis if they were recorded for at least 6 min after obtaining whole-cell access. To examine the influence of inhibiting the 5α-R enzyme, finasteride (50 μM) was pre-incubated with the cortical slice in a holding chamber containing oxygenated ECS (at room temperature) for > than 4 h prior to recording. Subsequent recordings from such slices were made either with a control intracellular pipette solution, or with the pipette containing γ-CD, to determine the combined influence of intracellular γ-CD and finasteride treatment. Note the final DMSO concentration (0.1%) had no effect on any of the mIPSC parameters measured.

### 2.5 Electrophysiological analysis

Digitized data was analysed offline using WinEDR/WinWCP software (Strathclyde University, UK). The mIPSCs were identified by an algorithmic detection protocol. To eliminate distal events, which may be affected by imperfect voltage-clamp, Gaussian distributions of 10–90% rise time were generated and mIPSCs falling outside the Gaussian limits were excluded. Individual mIPSCs were visually inspected and spurious events omitted. Typically, for each neuron data from 50, or more mIPSCs were analysed with respect to their peak amplitude, 10–90% rise time, and time taken to decay from peak by 50% (T50). Accepted mIPSCs recorded from a single neuron were averaged and fitted with either a mono-exponential (y(t) = Ae^(−t/τ)^), or bi-exponential (y(t) = A_1_e^(−t/τ1)^ + A_2_e^(−t/τ2)^) decay function, where y(t) is the current amplitude at time t, A is the current amplitude and τ is the decay time constant. To compare goodness of fit between a mono- or bi-exponential decay, an F test was applied to the standard deviation of the residuals. The overwhelming majority of mIPSC decay times analysed were best fit by a bi-exponential function. Subsequently, a mean weighted decay constant (τ_w_) was calculated to accommodate the relative contribution of each decay component whereby:

τ_w_ = τ_1_P_1_+τ_2_P_2_

Here, τ_1_ and τ_2_ are the decay time constants for the first and second exponential functions, and P_1_ and P_2_ are the proportions of current amplitude described by each component *i.e*.

$$\text{P}1=\frac{A1}{A1+A2}\;\text{P}2=\frac{A2}{A1+A2}$$

All reported data are expressed as mean values ± standard error of the mean (S.E.M.). To determine statistical significance, Student's t-tests (paired, or unpaired) and ANOVA (one or two-way, followed *post-hoc* by Tukey's HSD or independent samples t-test, SigmaStat, Systat Software Inc. San Jose, CA, USA) were used as appropriate. For comparison of cumulative probability distributions of mIPSC T50 values, the Kolmogorov-Smirnoff (KS) test was used (SPSS software, Chicago, IL, USA).

## 3 Results

### 3.1 The influence of development on phasic currents, mediated by synaptic GABA_A_Rs of L2/3 cortical pyramidal neurons

**Fig. 1:**
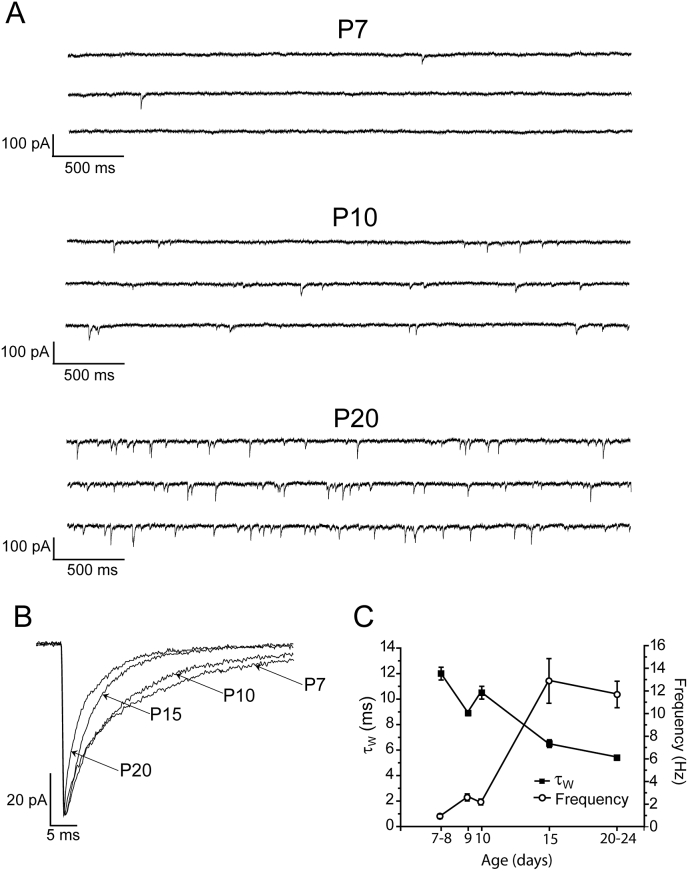
**The properties of mIPSCs recorded from WT L2/3 pyramidal neurons during postnatal development. A)**. Traces showing typical current recordings from L2/3 pyramidal neurons derived from WT mice at P7 (top), P10 (middle) and P20 (bottom). Note the increase in the frequency of synaptic events with development. **B)**. Averaged, superimposed mIPSCs normalised with respect to peak amplitude, recorded from representative WT L2/3 pyramidal neurons of P7, P10, P15 and P20 mice. The decay time decreases progressively with development. **C)**. A graph showing both the net decrease in τ_W_, and the concomitant increase in mIPSC frequency (n = 14–55 neurons) occurring with development. Symbols represent the mean ± S.E.M.

The properties of mIPSCs (frequency, amplitude and kinetics), recorded from WT L2/3 pyramidal neurons, obtained from neonatal/juvenile (P7 - 15) to adolescent (P20 - 24) mice, changed with development (Fig. 1; Table 1). Of particular note, with age the mIPSC frequency increased considerably (*e.g.* P7 - 8 = 1.2 ± 0.2 Hz, n = 55 neurons; P20 - 24 = 11.7 ± 1.2 Hz, n = 25 neurons – Fig. 1; Table 1). Furthermore, the mIPSC decay time, as quantified by determination of the weighted decay time constant (τ_W_), decreased with development. Specifically, P7 - 8 neurons exhibited mIPSCs with a relatively prolonged decay (τ_W_ = 12.1 ± 0.3 ms; n = 55), that by P15 had become significantly reduced (τ_W_ = 6.5 ± 0.3 ms; n = 14; one way ANOVA; p < 0.001 *vs* P7 - 8, Fig. 1C, Table 1). With the profile of mIPSC decay kinetics between P7 - 8 and P20 - 24 established, investigations now focused on whether the mIPSCs of L2/3 pyramidal neurons are influenced by endogenous neurosteroids.

### 3.2 Phasic GABAergic transmission from P7 - 8 cortical L2/3 pyramidal neurons is influenced by an endogenous neurosteroid tone

**Fig. 2:**
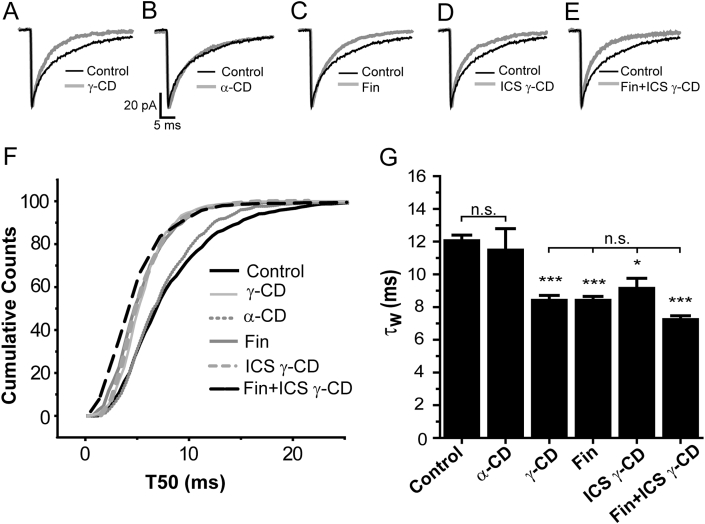
**GABAergic synaptic currents of L2/3 pyramidal neurons are influenced by an endogenous neurosteroid tone during early postnatal development. A - E)**. Averaged mIPSCs, superimposed and normalised with respect to peak amplitude from a representative P7 cortical pyramidal neuron during control conditions (black line) and following treatment (grey lines) with either, **A**) 1 mM γ-CD, **B**) 1 mM α-CD, **C**) 50 μM finasteride **(**Fin**)**, **D**) 0.5 mM γ-CD in the ICS only, or **E**) 50 μM finasteride + 0.5 mM γ-CD in the ICS. **F)**. A cumulative probability plot of the mIPSC T50 for control P7 - 8 L2/3 pyramidal neurons (events pooled from 55 cells) and P7 - 8 L2/3 pyramidal neurons following treatment conditions described above. All treatments apart from α-CD resulted in a significant leftward shift in the T50 distribution indicating that these treatments resulted in mIPSCs with reduced decay times compared with control (in all cases p < 0.001, KS-test). **G)** Summary bar graph depicting the mean τ_w_ values for P7 - 8 mIPSCs for control and after cyclodextrin/finasteride treatments (n = 6–55 cells). * = p < 0.05, *** = p < 0.001, *vs* control. n.s. = not significant, one way ANOVA with Tukey *post hoc* test.

To test for endogenous modulation of GABAergic neurotransmission by neurosteroids, we utilized γ-CD, a neurosteroid scavenger (Shu et al., 2007, Brown et al., 2015). For P7 - 8 neurons, the γ-CD pre-incubation protocol (>1 h. see Methods) had no effect on the mIPSC frequency, or amplitude (in both cases p > 0.05, one way ANOVA), but greatly reduced their duration (τ_W_ control = 12.1 ± 0.3 ms, n = 55, *vs* τ_W_ γ-CD = 8.5 ± 0.2 ms, n = 20, p < 0.001, one way ANOVA, Fig. 2A, F, G Table 1). The structurally related α-CD is ineffective in sequestering pregnane steroids, as the pore diameter of the molecule is smaller (6 *vs* 8 cyclic sugars) than that of γ-CD (Davis and Brewster, 2004, Shu et al., 2004, Shu et al., 2007, Brown et al., 2015). Importantly, the equivalent treatment with α-CD had no effect on the mIPSC τ_W_ of P7 - 8 neurons (p > 0.05, one way ANOVA, Fig. 2B, F, G).

To confirm that the reduced decay times observed following γ-CD were due to neurosteroid sequestration, we pre-incubated P7 - 8 WT cortical brain slices with the 5α-reductase inhibitor finasteride (50 μM > 4 h), which significantly reduced the mIPSC duration (τ_W_ control = 12.1 ± 0.3 ms, n = 55; τ_W_ finasteride = 8.5 ± 0.3 ms, n = 7, p < 0.001, one way ANOVA, Fig. 2C, F, G, Table 1). This effect on the mIPSC duration was indistinguishable from that produced by γ-CD (finasteride τ_W_ = 8.5 ± 0.3 ms, n = 7, γ-CD τ_W_ = 8.5 ± 0.2 ms, n = 20; p > 0.05, one way ANOVA, Fig. 2F, G, Table 1). Collectively, these findings indicate that an endogenous neurosteroid tone influences synaptic GABA_A_Rs at P7 – 8.

Interpretation of the combined effects of finasteride and γ-CD is potentially complicated, as given that finasteride is a steroid, it is conceivable that the extracellular γ-CD may sequester this 5α-reductase inhibitor. We have previously demonstrated that intracellular γ-CD alone is equi-effective in influencing the mIPSCs of developing thalamic neurons (Brown et al., 2015), suggesting a protocol to avoid this complexity. Therefore, we first investigated the effect on cortical mIPSCs of incorporating the membrane-impermeant γ-CD (0.5 mM) solely in the recording pipette (ICS γ-CD). This treatment (recordings made > 6 min after achieving the whole-cell recording configuration) significantly reduced the mIPSC τ_W_ (control = 12.1 ± 0.3 ms, n = 55; γ-CD ICS: 9.2 ± 0.6 ms, n = 6, p < 0.05, one way ANOVA, Fig. 2D, F, G), an effect indistinguishable from that of γ-CD resulting from the pre-incubation protocol (γ-CD pre-incubation: 8.5 ± 0.2 ms, n = 20, p > 0.05, one way ANOVA, Fig. 2F, G) and not significantly different from that produced by finasteride (50 μM) treatment (p > 0.05, one way ANOVA τ_W_ = 8.5 ± 0.3 ms, n = 7, Fig. 2F, G). Finally, we now determined the combined effect of finasteride and γ-CD treatment. For P7 - 8 neurons, treatment of the slice with finasteride (50 μM) for > 4 h, followed by intracellular γ-CD (0.5 mM), resulted in mIPSCs with a significantly reduced duration (τ_w_ = 7.3 ± 0.2 ms; n = 6, p < 0.001, one way ANOVA), that was not significantly different from that produced solely by finasteride, or by intracellular γ-CD alone (p > 0.05, one way ANOVA, Fig. 2E, F, G).

### 3.3 The neurosteroid influence on phasic–GABAergic transmission of cortical L2/3 pyramidal neurons changes during development

**Fig. 3:**
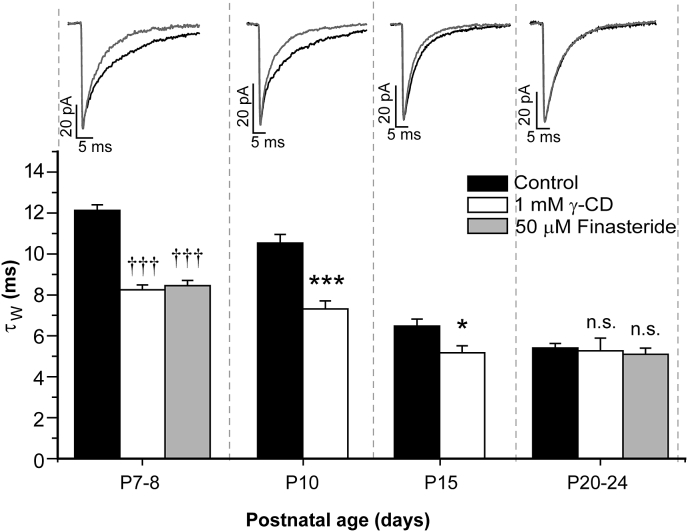
**The cortical neurosteroid tone is developmentally regulated**. A bar graph showing the effect of γ-CD pre-incubation (1 mM) on the τ_W_ of mIPSCs recorded from WT L2/3 pyramidal neurons during development (n = 8–55 cells). Note that at P7 - 8, P10 and P15 the γ-CD pre-incubation resulted in a significant reduction in τ_W_*vs* control. For comparison, the effect of finasteride (50 μM) preincubation at P7 - 8 and P20 - 24 is also shown (n = 6–55 cells). ††† = p < 0.001, *vs* control, *post hoc* Tukey HSD test following two-way ANOVA. * = p < 0.05, *** = p < 0.001, *vs* control, *post-hoc* independent samples t-test following two-way ANOVA. n.s. = not significant. The independent variables for the two-way ANOVAs were postnatal age and treatment (γ-CD, or finasteride). Illustrated above each developmental time point are the corresponding averaged and superimposed mIPSCs, normalised with respect to peak amplitude, obtained from representative pyramidal neurons in the absence (control, black line) and following 1 mM γ-CD pre-incubation (grey line).

We next assessed whether neurosteroids contribute to the developmental changes in the duration of phasic GABAergic events by determining the effect of γ-CD on the mIPSCs of neurons at different stages of development (Brown et al. 2015). Treatment with γ-CD reduced the decay time of mIPSCs recorded from P7 - 8, P10 and P15 neurons, relative to their respective controls (Fig. 3, Table 1). However, the developmental stage significantly influenced the effect of γ-CD on the mIPSC decay time (Fig. 3, age × treatment interaction, F_3,157_ = 6.15, p < 0.001, two-way ANOVA), such that by P20-24, γ-CD had no significant effect (control τw = 5.4 ± 0.2 ms n = 25; γ-CD = 5.3  ± 0.6 ms; n = 8; p > 0.05; independent samples t-test, Fig. 3). Further implicating a changing neurosteroid impact during development, the effect of finasteride (50 μM) was also significantly influenced by post-natal age (Fig. 3, age × treatment interaction, F_2,127_ = 10.47, p < 0.001, two-way ANOVA), such that, in contrast to P7 – P8 recordings, finasteride had no significant effect on the mIPSC duration of P20 – P24 neurons (control τ_W_ = 5.4 ± 0.2 ms; n = 25; finasteride τ_W_ = 5.3 ± 0.2 ms; n = 6; p > 0.05, post-hoc Tukey HSD, Fig. 3).

### 3.4 Decreased neurosteroid synthesis contributes to the changes to phasic GABAergic transmission evident in P20-24 cortex

**Fig. 4:**
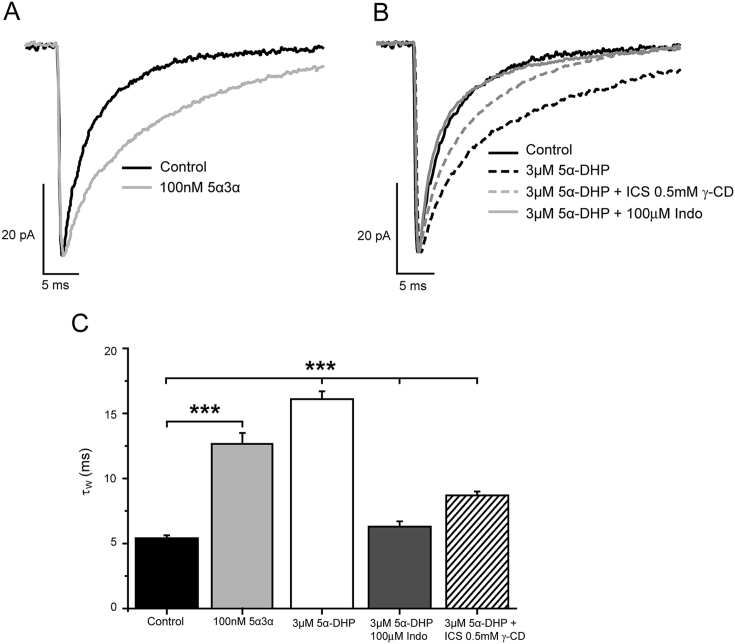
**The synaptic GABA**_**A**_**Rs of P20 – 24 L2/3 cortical pyramidal neurons are neurosteroid sensitive and furthermore, P20 – 24 cortical brain slices can synthesise GABA**_**A**_**R-active neurosteroids. A)**. Averaged, superimposed mIPSCs normalised with respect to peak amplitude, recorded from representative WT L2/3 cortical pyramidal neurons from brain slices derived from P20 - 24 mice, under control conditions and following incubation (>120 min) with 100 nM 5α3α. **B)** Averaged, superimposed P20-24 mIPSCs normalised with respect to peak amplitude, recorded under control conditions and following incubation (>120 min) with 3 μM 5α-DHP; co-incubation with 100 μM indomethacin (Indo) and 3 μM 5α-DHP (>120 min); or incubation with 3 μM 5α-DHP (>120 min) followed by 0.5 mM ICS γ-CD. **C)** Summary bar graph illustrating the increase in the mIPSC τ_w_ in response to incubation of 100 nM 5α3α; 5α-DHP incubation and the much reduced 5α-DHP effect following either co-incubation with 100 μM indomethacin (Indo), or addition of 0.5 mM γ-CD to the ICS (n = 5–25 cells). *** = p < 0.001 *vs* control, one way ANOVA with Tukey *post hoc* analysis.

The loss of neurosteroid influence on phasic inhibition of P20 - 24 pyramidal neurons, inferred by both the finasteride and γ-CD experiments, may be due to the synaptic GABA_A_Rs becoming neurosteroid-insensitive (Koksma et al. 2003), or alternatively a consequence of a decreased neurosteroid synthesis. The neurosteroid interaction with GABA_A_Rs may be influenced by factors such as subunit composition and phosphorylation status (Koksma et al., 2003, Belelli and Lambert, 2005). We therefore investigated whether GABA_A_Rs expressed by P20 - 24 L2/3 pyramidal neurons retained neurosteroid sensitivity. The acute bath application of exogenous 5α3α (1 μM, whereby mIPSCs were analysed before, and after ∼ 7 min application of the steroid) resulted in mIPSCs with a significantly prolonged decay phase (control τ_W_: 5.5 ± 0.5 ms *vs* 1 μM 5α3α τ_W_: 8.7 ± 1.7 ms, n = 7, p < 0.05, paired t-test). For isolated single cell studies, acutely applied 5α3α acts at nM aqueous concentrations to enhance GABA_A_R function (Pistis et al., 1997, Belelli and Lambert, 2005). Therefore, the relatively limited effect of acutely applied 5α3α at the relatively high concentration of 1 μM might suggest that these cortical synaptic GABA_A_Rs are relatively insensitive to the neurosteroid by P20-24. Alternatively, the effect of the steroid when applied acutely to a brain slice may be underestimated. In support of the latter, the general anesthetics etomidate and propofol, which in common with neurosteroids are lipophilic and efficacious GABA_A_R modulators, require several hours to approach equilibrium within *in vitro* brain slice preparations (Gredell et al., 2004, Benkwitz et al., 2007). To ascertain whether the GABA modulatory effects of 5α3α are underestimated when applied acutely to a cortical slice, we determined the effect of a lower concentration (100 nM) of 5α3α on the mIPSCs of P20-24 L2/3 pyramidal neurons, but now pre-incubated (>2 h), before being continuously applied during the recording. Employing this protocol, the 10 fold lower concentration of 5α3α (100 nM) produced a clear and large prolongation of the mIPSC decay (control τ_W_ = 5.4 ± 0.2 ms, n = 25 *vs* 5α3α 100 nM τ_W_ = 12.6 ± 0.9 ms, n = 5, p < 0.001, one way ANOVA*; post-hoc* Tukey HSD Fig. 4A, C). Importantly, this experiment establishes that P20 - 24 cortical synaptic receptors retain sensitivity to nM aqueous concentrations of this neurosteroid and consequently, the change in mIPSC decay at this stage of development is not due to neurosteroid-insensitive synaptic GABA_A_Rs.

To determine whether more mature (P20 - 24) L2/3 cortical pyramidal neurons retain the capacity to synthesize GABA_A_R-active neurosteroids, we investigated the influence of the GABA_A_R-inactive steroid 5α-DHP, the immediate precursor of 5α3α (Brown et al. 2015). We had previously shown that a prolonged incubation (>2 h), but not a short incubation (30–60 min) of thalamic slices with 5α-DHP prolonged the mIPSC decay phase of VB neurons (Brown et al. 2015). Here, pre-incubation of the cortical slice with 5α-DHP (3 μM) for > 2 h, followed by continuous perfusion of this steroid during the recording (see Methods), resulted in greatly prolonged mIPSCs (control τ_W_ = 5.4 ± 0.2 ms, n = 25 *vs* 5α-DHP τ_W_ = 16.1 ± 0.6 ms, n = 7, p < 0.001, one way ANOVA - see Fig. 4B, C). This effect was markedly reduced by co-incubation with the 3α-HSD inhibitor indomethacin (100 μM; τ_W_ = 6.3 ± 0.4 ms; n = 6; *post hoc* Tukey HSD p < 0.001), or by intracellular (0.5 mM) γ-CD (τ_W_ = 8.7 ± 0.3 ms; n = 6, *post hoc* Tukey HSD p < 0.001) - Fig. 4B, C. Therefore, when provided with the immediate precursor, cortical tissue from P20-24 mice retains the ability to synthesize GABA_A_R-active neurosteroids. Furthermore, these data provide additional evidence that their synaptic GABA_A_Rs remain neurosteroid sensitive.

### 3.5 Phasic GABAergic transmission of L2/3 pyramidal neurons is influenced both by an endogenous neurosteroid tone and by the subunit composition of synaptic GABA_A_Rs

**Fig. 5:**
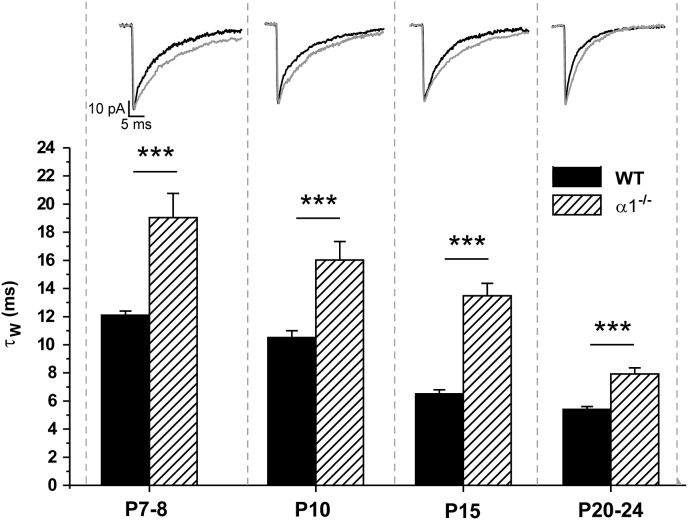
**The effect of α1 subunit deletion on the τ**_**W**_**of mIPSCs recorded from L2/3 neurons during development**. A bar graph comparing τ_W_ of mIPSCs recorded from WT and α1^−/−^ L2/3 neurons during postnatal development (n = 4–13 α1^−/−^ neurons). The WT data is adapted from Fig. 1. Note that the decay time of mIPSCs of both WT and α1^−/−^ L2/3 pyramidal neurons, becomes reduced with age. However, at each developmental stage, mIPSCs recorded from α1^−/−^ neurons exhibit a slower decay *c.f*. those recorded from equivalent WT neurons (*** = p < 0.001, *post-hoc* independent samples t-test following two-way ANOVA, with postnatal age and genotype as the independent variables). Illustrated above each developmental time point are corresponding averaged and superimposed mIPSCs, normalised with respect to peak amplitude, obtained from representative WT (black line) and α1^−/−^ pyramidal neurons (grey line).

**Fig. 6:**
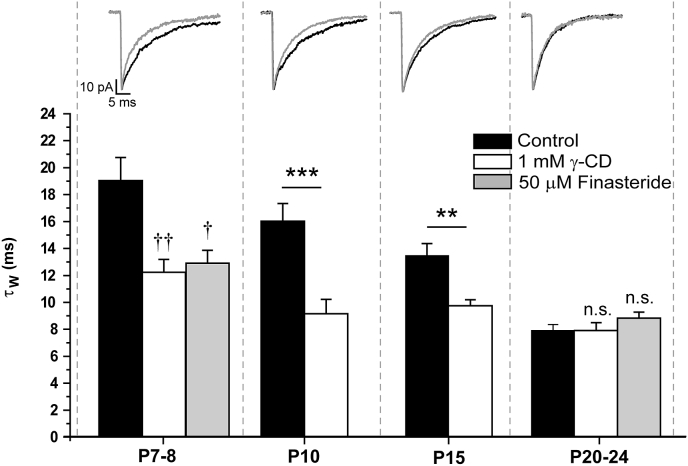
**A developmentally-regulated neurosteroid tone influences the decay time of mIPSCs recorded from α1**^**−/−**^**L2/3 neurons**. A bar graph showing the effect of γ-CD pre-incubation (1 mM) on the τ_W_ of mIPSCs recorded from α1^−/−^ L2/3 pyramidal neurons during development (n = 4–13 cells). In common with WT L2/3 pyramidal neurons, treatment of α1^−/−^ cortical brain slices with γ-CD results in faster decaying mIPSCs at P7 - 8, P10 and P15, but not at P20-24. For comparison, the effect of finasteride (50 μM) pre-incubation at P7 - 8 and P20 - 24 is also shown (n = 7–14 cells). †† = p < 0.01, † = p < 0.05 *vs* control, *post hoc* Tukey HSD test following a two-way ANOVA. ** = p < 0.01, *** = p < 0.001, *vs* control, *post-hoc* independent samples t-test following a two-way ANOVA. n.s. = not significant. The independent variables for the two-way ANOVAs were postnatal age and treatment. Illustrated above each developmental time point is the corresponding averaged and superimposed mIPSCs, normalised with respect to peak amplitude, obtained from representative pyramidal neurons of WT and α1^−/−^ in the absence (control, black line) and following 1 mM γ-CD pre-incubation (grey line).

Despite treatment with γ-CD, the mIPSC decay time still decreased with development, with a similar trend observed for finasteride-treated neurons (Fig. 3, Table 1). These observations suggest that factors additional to neurosteroids influence phasic inhibition during development. Numerous studies have implicated changes to the subunit composition of synaptic GABA_A_Rs to be important in this respect, with a particular emphasis on the role of the α1 subunit (Rovira and Ben-Ari, 1993, Tia et al., 1996, Hollrigel and Soltesz, 1997, Dunning et al., 1999, Kapur and Macdonald, 1999, Vicini, 1999, Vicini et al., 2001, Okada et al., 2000, Ortinski et al., 2004). To investigate whether α1-GABA_A_Rs influence mIPSCs during the development (P7 – 24) of L2/3 pyramidal neurons, cortical brain slices were prepared from mice engineered to lack the α1 subunit (α1^−/−^). The decay phase of α1^−/−^ mIPSCs was prolonged in comparison to their WT counterparts, but, importantly, this occurred at all ages examined here (two-way ANOVA, age × genotype interaction, F_3,139_ = 5.75, p = 0.001; for *post-hoc* WT *vs* α1^−/−^ comparisons, p < 0.001 for P7 - 8, P10, P15 and P20, Fig. 5). However, in common with WT neurons, the α1^−/−^ mIPSC decay phase became faster with development (p < 0.001, one way ANOVA, Fig. 5; Table 2), suggesting that factors other than increased expression of α1-subunit containing GABA_A_Rs must contribute to the developmental profile. In agreement, and further implicating a role for neurosteroids, γ-CD treatment significantly reduced the decay time of α1^−/−^ mIPSCs in P7 - 8, P10 and P15 α1^−/−^ neurons, but in common with their WT counterparts, had no effect on the τw of P20 - 24 α1^−/−^ mIPSCs (two-way ANOVA, age × treatment interaction, F_3, 58_ = 6.14, p = 0.001; for *post-hoc* control vs γ-CD comparisons, p < 0.01 for α1^−/−^ P7 - 8, P10 and P15 neurons and p > 0.05 for P20-24, Fig. 6). Similarly, finasteride (50 μM) treatment reduced the τ_W_ of P7 - 8 α1^−/−^ mIPSCs, but had no such effect on P20 - 24 α1^−/−^ mIPSCs (two-way ANOVA, age × treatment interaction F_2,43_ = 9.87, p < 0.001; for *post-hoc* control *vs* finasteride comparisons, p < 0.05 for P7 – 8 and p > 0.05 for P20-24; Fig. 6, Table 2). These observations suggest that the duration of cortical mIPSCs is influenced throughout the developmental period studied here by the expression of synaptic receptors incorporating the α1 subunit, but that changes to the expression of α1-GABA_A_Rs are not exclusively responsible for the altered mIPSC kinetics occurring within this developmental window. Furthermore, in common with WT, the waning of a neurosteroid tone is revealed to be an important determinant of the duration of α1^−/−^ mIPSCs.

### 3.6 The role of neurosteroids in mediating the developmental changes to phasic GABAergic transmission of L2/3 cortical interneurons

**Fig. 7:**
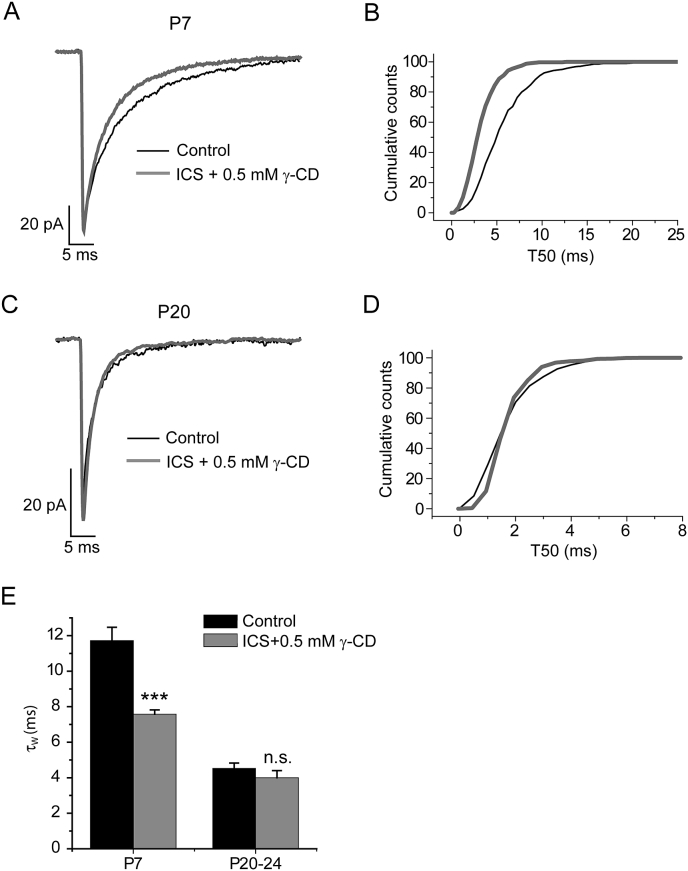
**The effect of intracellular γ-cyclodextrin (0.5 mM) on the decay kinetics of mIPSCs recorded from P7 and P20 - 24 L2/3 cortical GAD67-GFP + neurons. A, C)**. Superimposed, averaged mIPSCs normalised with respect to peak amplitude from representative P7 (**A**) and P20 (**C**) control L2/3 GFP + neurons (black line) and L2/3 GFP + neurons in which the intracellular solution contained 0.5 mM γ-CD (grey line) at P7 (**A**) and P20 (**C**). **B, D)**. Cumulative probability plots of the mIPSC T50 for P7 (**B**) and P20 - 24 neurons (**D**) control (black line) and ICS + γ-CD (grey line). In each case events were pooled from n = 8 GFP + cells (P7 control), n = 8 GFP + cells (P7 ICS+ γ-CD), n = 14 GFP + cells (P20 - 24 control), and n = 7 GFP + cells (P20 - 24 ICS+ γ-CD). For P7, but not for P20 - 24 cells, the mIPSC T50 distribution is left-shifted indicating that all mIPSCs recorded from P7 GFP + cells treated with intracellular γ-CD, exhibited faster decay kinetics, compared with control (p < 0.001, KS-test). **E)**. A summary bar graph showing a significant decrease in the mIPSC τ_W_ following intracellular γ-CD treatment at P7, but not at P20 - 24. n.s. = not-significant; *** = p < 0.001 *vs* control, *post-hoc* independent samples t-test following two-way ANOVA, with postnatal age and treatment as the independent variables.

We next investigated whether the developmentally regulated neurosteroid tone is specific for L2/3 pyramidal neurons, or is more generally experienced by other neuronal populations. To identify GABA-ergic interneurons we utilized GAD 67 GFP + mice, engineered to co-express green fluorescent protein (GFP) with the GABA-synthesising 67 kDa γ-amino decarboxylase (GAD 67) enzyme (Tamamaki et al. 2003). Co-localization studies revealed that three major interneuron classes present in mouse neocortex, (*i.e*. calretinin-, parvalbumin-, or somatostatin-expressing) are all GFP-positive (Tamamaki et al. 2003). Using epifluorescence microscopy, recordings from P7 - 8 GFP expressing neurons of L2/3, revealed mIPSCs with a decay phase (τ_W_ = 11.7 ± 0.8 ms, n = 8; Fig. 7 A, B, E - see Table 3 for additional properties), which at this age is similar to that of pyramidal neurons (P7 - 8 L2/3 pyramidal τ_W_ = 12.1 ± 0.3 ms, n = 55, p > 0.05, unpaired t-test), of WT mice. In common with cortical pyramidal neurons, the mIPSC properties of GABA-ergic interneurons changed with development (Table 3). In particular, P20 - 24 interneuron mIPSCs exhibited a much reduced decay time (τ_W_ = 4.5 ± 0.3 ms, n = 14, p < 0.001, unpaired t-test, Fig. 7 C, D, E, Table 3), compared to their younger counterparts.

For P7 - 8 GABA-ergic interneurons, intracellular γ-CD (0.5 mM) had no effect on either the mIPSC peak amplitude, rise time, or frequency (in all cases p > 0.05 *vs* control, unpaired t-test), but greatly reduced their decay time (P7 - 8 τ_W_ control = 11.7 ± 0.8 ms, n = 8, τ_W_ ICS γ-CD = 7.6 ± 0.2 ms, n = 8, p < 0.001, unpaired t-test, Fig. 7 A, B, E, Table 3). However, the effect of intracellular γ-CD was significantly influenced by developmental stage (two-way ANOVA, age × treatment interaction, F_1,33_ = 16.23, p < 0.001), such that the mIPSC decay of P20 - 24 interneurons was no longer influenced by the steroid scavenger (τ_W_ control = 4.5 ± 0.3 ms, n = 14; ICS γ-CD τ_W_ = 4.0 ± 0.4, n = 7, p > 0.05, unpaired t-test, Fig. 7 C, D, E, Table 3). Therefore, phasic inhibition of L2/3 interneurons, in common with pyramidal neurons, changes with development. Furthermore, early in development a neurosteroid tone is experienced by the synaptic GABA_A_Rs of both GABA-ergic interneurons and principal neurons, which by P20 - 24 dissipates, resulting in brief phasic inhibitory events.

## 4 Discussion

### 4.1 Endogenous neurosteroids prolong the mIPSCs of cortical L2/3 neurons during development

Postnatal development is marked by periods of considerable plasticity within cortical circuitry, wherein GABAergic neurotransmission is driven towards rapid and effective phasic inhibition, capable of supporting the complexity of mature cortical processing. During this period, several mechanisms may contribute to the mIPSC decay time, including: alterations in the subunit composition of synaptic GABA_A_Rs (Takahashi, 2005, Eyre et al., 2012), post-translational modifications of synaptic proteins (Vithlani et al. 2011), the extent of receptor clustering (Petrini et al. 2003), changes in the kinetics of GABA release and alterations to GABA uptake (Mozrzymas, 2004). The latter is influenced by the activity and location of the various GABA transporters. However, whereas the effects of transporter inhibitors on the time course of responses to iontophoretically applied GABA, or on IPSCs evoked by repetitive nerve stimulation are quite evident, they have relatively little effect on the amplitude or kinetics of mIPSCs (Keros and Hablitz, 2005, Scimemi, 2014). Our results indicate that in addition to a possible involvement of such factors, during postnatal development the mIPSCs of L2/3 pyramidal neurons become reduced in duration, at least in part due to a programmed loss of the influence of endogenous neurosteroids upon synaptic GABA_A_Rs. The validity of our conclusions is partly dependent on the specificity of γ-CD in sequestering neurosteroids. A previous study reported that β-CD (0.5–1.5 mM), when applied to hippocampal neurons prolonged the decay of macroscopic currents mediated by GABA_A_Rs (Pytel et al. 2006). However, γ-CD treatment of thalamic VB neurons, cortical L2/3 interneurons and pyramidal cells induced a marked reduction in the mIPSC decay time early in development, but had no effect on this parameter at later developmental time-points (*e.g.* P20-24; Brown et al., 2015). Furthermore, pre-incubation with α-CD had no effect on any of the mIPSC properties at any age studied here and most importantly the effects of γ-CD on P7-8 neurons were recapitulated by pre-incubation with the 5α−R inhibitor finasteride. A parsimonious explanation for the decreased duration of immature L2/3 mIPSCs following γ-CD treatment posits that the steroid-sequestering molecule is effective in forming inclusion complexes with endogenous neurosteroids, whereas the observed insensitivity to α-CD reflects the hydrophobic inner cavity being too small to accommodate steroids (Szejtli, 1998, Shu et al., 2004, Brown et al., 2015).

Theoretically, the loss of neurosteroid influence on phasic inhibition with development may result from the synaptic GABA_A_Rs becoming insensitive to this endogenous modulator (Koksma et al. 2003). However, acute application of 5α3α (1 μM) clearly prolonged mIPSCs recorded from P20-24 cortical L2/3 pyramidal neurons. Furthermore by pre-incubating the tissue with a lower, aqueous concentration (100 nM) of 5α3α, we demonstrated these synaptic GABA_A_Rs to be highly sensitive to the neurosteroid at this stage of development. Alternatively, a change in steroid enzyme expression, or a lack of steroid substrate(s) may be implicated in this developmental plasticity. We previously demonstrated that incubation of mouse thalamic slices with 5α-DHP, the immediate precursor of 5α3α, greatly increased the duration of the mIPSCs of VB neurons (Brown et al. 2015). Similarly here, incubation of P20 - 24 cortical tissue with 5α-DHP greatly prolonged the mIPSCs of cortical pyramidal neurons and in common with thalamic neurons, this effect was prevented by co-incubation with the 3α-HSD inhibitor indomethacin, or reversed by intracellular γ-CD (Brown et al. 2015). Collectively these results suggest that the developmental change to phasic inhibition of cortical pyramidal neurons occurring between P7 and P20 results in a part from a lack of steroid substrate.

### 4.2 Location of neurosteroid synthesis and action

Previous histochemical studies support the concept of a local neurosteroid synthesis in cortex. In mouse cortex the staining for mRNA encoding for the 5α3α synthesising enzymes 5α-reductase Type I (5α-R I) and 3α-hydroxysteroid dehydrogenase (3α-HSD), was co-located in layer 2/3/5 pyramidal neurons (Agis-Balboa et al. 2006). The 5α-R I staining co-localised with that for the vesicular glutamate transporter (VGLUT1), a marker of glutamatergic neurons (Agis-Balboa et al. 2006). By contrast, the 5α-R I, or the 3α-HSD staining did not co-localize with a marker for GABA-ergic neurons, or for glia (Agis-Balboa et al. 2006). In apparent agreement, an antibody raised against 5α3α revealed staining for this GABA_A_R-active steroid in rat cortical L2-6 pyramidal neurons, but not in cells that had the appearance of GABA-ergic interneurons, or glia, but note were not categorically identified by specific neurochemical markers of interneuron subtypes (Saalmann et al. 2007). However, a recent study identified expression of 5αR Type II in cortical GABAergic cells, suggesting that neurosteroid synthesis and action may not always be confined to principal excitatory neurons (Castelli et al. 2013).

Whether cortical principal cells or interneuron populations are the locus of neurosteroid synthesis is not directly addressed by our finasteride, or intracellular γ-CD experiments. If at P7 - 8 the mode of neurosteroid action is exclusively autocrine, then a ubiquitous expression of steroid-synthesizing enzymes across different neuronal populations would be required. Alternatively, the local steroid concentration present during neonatal development may be sufficient to impact upon GABA-ergic and/or principal neurons, which are incapable of neurosteroid synthesis, thereby inferring a paracrine mode of action. Clearly, further studies are required to clarify the relative contribution of autocrine and paracrine neurosteroids to the inhibitory plasticity of the developing cortex.

Irrespective of the locus of synthesis, the lipophilic steroid is considered to access the synaptic GABA_A_Rs by lateral diffusion *via* the plasma membrane, a mechanism congruent with the proposed transmembrane neurosteroid binding site on the receptor (Hosie et al., 2006, Chisari et al., 2010). Numerous *in vitro* electrophysiological studies report enhancement of GABA_A_R function by low nM aqueous concentrations of 5α3α (see Belelli and Lambert, 2005), advocating the presence of a relatively high affinity binding site on the GABA_A_R. However, such neurosteroids are highly lipophilic, permitting much greater local concentrations to accumulate in the vicinity of the receptor, obviating the requirement for a high affinity binding site. Indeed, the proposed low affinity binding site (Chisari et al. 2010) is consistent with our observation that even when γ-CD was applied exclusively to the cytosolic compartment, it efficiently removed the neurosteroid influence on synaptic GABA_A_Rs of immature cortical pyramidal neurons.

### 4.3 The role of GABA_A_R subunit composition in developmental plasticity of phasic GABAergic neurotransmission

The α1 subunit mRNA and protein is present in the cortex early in development, albeit at low levels, whereas the converse is true for α2/3 subunits, which are highly expressed early in life, before they decrease to lower levels in the mature cortex (Laurie et al., 1992, Fritschy et al., 1994, Pirker et al., 2000). Here the mIPSCs obtained from α1^−/−^ L2/3 pyramidal neurons at P7 - 8, P10, P15 and P20 - 24 exhibited slower decay kinetics *c.f.* WT controls at each developmental time-point. Since α1-GABA_A_Rs are associated with fast decay kinetics (Picton and Fisher, 2007), this finding suggests that a proportion of synaptic α1-GABA_A_Rs are present even at P7 - 8 in L2/3 pyramidal neurons. However, a developmental decrease of the mIPSC decay time of α1^−/−^ L2/3 pyramidal neurons was still evident, implying the presence of additional factors, as previously suggested (Bosman et al. 2005). Moreover, the developmental profile of γ-CD sensitivity for mIPSCs from P7 - 8, P10 and P15 α1^−/−^ L2/3 pyramidal neurons was indistinguishable to WT. In summary, these findings indicate that although the duration of the mIPSCs is influenced by the α1 subunit, it is not solely responsible for the developmental changes that occur in the postnatal period P7 – 20. The results presented here reveal the waning impact during development of the endogenous neurosteroid tone is an additional important factor in influencing phasic GABAergic neurotransmission of both WT and α1^−/−^ L2/3 pyramidal neurons. However, a comparison of the mIPSC decay of P7 - 8 and P20 - 24 α1^−/−^ neurons, when treated with either finasteride, or γ-CD, reveals an additional, as yet unidentified factor(s) that influences phasic GABAergic neurotransmission during postnatal development.

### 4.4 The physiological role of GABA_A_R-active neurosteroids during development

This study focused on L2/3 cortical neurons and found that the synaptic GABA_A_Rs expressed on both pyramidal and interneuron populations are developmentally influenced by neurosteroids to modulate the duration of GABAergic synaptic transmission. These changes in neurosteroid influence are occurring during an intense period of synaptogenesis, which may be reflected by our observation that the frequency of mIPSCs increases during postnatal development. Furthermore, at this time GABA may exert a depolarizing effect due to the dominance of the chloride importer NKCC1 (Owens et al., 1996, Ben-Ari et al., 2007). Hence, long duration synaptic events may be suited to recruiting voltage-gated calcium channel activation, which in turn may initiate various Ca^2+^-dependent processes in the neuron. In this scenario, the emergence of mature (hyperpolarizing) GABA-ergic signalling would inversely correlate with the decline of neurosteroid production. Adding complexity, a recent report has demonstrated that neonatal administration of 5α3α influences the hippocampal expression of the K^+^ Cl^−^ co-transporter KCC2 (Modol et al., 2014).

We recently reported a similar developmentally controlled neurosteroid tone in somatosensory thalamocortical neurons (Brown et al. 2015). During development, the window of neurosteroid influence on GABA-ergic transmission in the somatosensory thalamus is shorter than that described here for cortex. In thalamic neurons the neurosteroid tone was absent by P10 (Brown et al. 2015), whereas in cortex this form of endogenous modulation persisted through to P15, but was no longer present by P20 - 24. The reasons for the distinct temporal regulation between thalamus and cortex are unknown. In addition to thalamus and cortex, previous studies have identified a similar neurosteroid tone in spinal neurons of the dorsal horn (Keller et al. 2004), which also exhibits a differential developmental profile for lamina II and lamina III/IV neurons (Inquimbert et al. 2008). Hence, the existence of a neurosteroid tone at multiple tiers of the CNS suggests a widespread role during postnatal development. Deciphering the interplay between endogenous neurosteroid synthesis, GABA_A_R potentiation, and neuronal maturation should therefore be a focus for future studies.

## Figures and Tables

**Table 1:** **A summary of the impact of development and γ-CD, or finasteride preincubation on the mIPSC properties of WT L2/3 pyramidal neurons**.* = p < 0.05,*** = p < 0.001, *vs* control, unpaired t-test.^†††^ = p < 0.001, *vs* control, one way ANOVA with Tukey *post hoc* test.

	P7/8			P10		P15		P20-24		
Control (n = 55)	γ-CD (n = 20)	FIN (n = 7)	Control (n = 20)	γ-CD (n = 11)	Control (n = 14)	γ-CD (n = 12)	Control (n = 25)	γ-CD (n = 8)	FIN (n = 6)
Peak amplitude (pA)	−50 ± 3	−48 ± 3	−63 ± 3	−46 ± 2	−47 ± 4	−54 ± 3	−45 ± 1*	−42 ± 2	−42 ± 3	−50 ± 5
Rise time (ms)	0.6 ± 0.1	0.6 ± 0.1	0.5 ± 0.1	0.6 ± 0.1	0.6 ± 0.1	0.5 ± 0.1	0.5 ± 0.1	0.5 ± 0.1	0.5 ± 0.1	0.5 ± 0.1
τ_W_ (ms)	12.1 ± 0.3	8.5 ± 0.2^†††^	8.5 ± 0.3^†††^	10.5 ± 0.5	7.3 ± 0.4***	6.5 ± 0.3	5.2 ± 0.3*	5.4 ± 0.2	5.3 ± 0.6	5.3 ± 0.2
Frequency (Hz)	1.2 ± 0.2	1.2 ± 0.2	1.8 ± 0.4	2.2 ± 0.3	2.6 ± 0.5	12.9 ± 2.0	12.4 ± 2.8	11.7 ± 1.2	12.6 ± 3.3	9.1 ± 0.7

**Table 2:** **A summary of the impact of development and γ-CD pre-incubation on the properties of mIPSCs of α1**^**−/−**^**L2/3 pyramidal neurons**.** = p < 0.01,*** = p < 0.001, *vs* control, Student's unpaired t-test.^†^ = p < 0.05,^††^ = p < 0.01, *vs* control, one way ANOVA with Tukey *post hoc* analysis.

	P7-8			P10		P15		P20-24		
Control (n = 5)	γ-CD (n = 8)	FIN (n = 5)	Control (n = 4)	γ-CD (n = 4)	Control (n = 12)	γ-CD (n = 9)	Control (n = 13)	γ-CD (n = 11)	FIN (n = 7)
Peak amplitude (pA)	−57 ± 3	−52 ± 2	−60 ± 6	−49 ± 2	−51 ± 6	−41 ± 3	−39 ± 3	−49 ± 3	−47 ± 2	−57 ± 3.8
Rise time (ms)	0.5 ± 0.1	0.5 ± 0.1	0.5 ± 0.1	0.5 ± 0.1	0.5 ± 0.1	0.5 ± 0.1	0.5 ± 0.1	0.5 ± 0.1	0.5 ± 0.1	0.4 ± 0.1
τ_W_ (ms)	18.7 ± 2.0	12.2 ± 1.0^††^	12.9 ± 0.9^†^	16.0 ± 1.3	9.2 ± 1.1***	13.5 ± 0.9	9.8 ± 0.4**	7.9 ± 0.4	7.9 ± 0.6	8.8 ± 0.4
Frequency (Hz)	ND	ND	ND	0.8 ± 0.3	1.5 ± 0.3	3.9 ± 0.7	3.5 ± 1.0	12.5 ± 1.6	8.5 ± 1.2	ND

**Table 3:** **A summary of the effects of intracellular γ-CD treatment on the properties of mIPSCs of L2/3 GAD 67 GFP + neurons**.*** = p < 0.001, *vs* control, unpaired t-test.

	P7 control (n = 8)	P7 γ-CD (n = 8)	P20-24 control (n = 14)	P20-24 γ-CD (n = 7)
Peak amplitude (pA)	−74 ± 7	−61 ± 5	−62 ± 4	−59 ± 7
Rise time (ms)	0.4 ± 0.1	0.4 ± 0.1	0.4 ± 0.1	0.4 ± 0.1
τ_W_ (ms)	11.7 ± 0.8	7.6 ± 0.2***	4.5 ± 0.3	4.0 ± 0.4
Frequency (Hz)	0.3 ± 0.1	0.2 ± 0.1	2.7 ± 0.5	2.1 ± 0.9
